# Legal Strategies Countering Federal Public Health Data Purges

**DOI:** 10.1017/jme.2025.10120

**Published:** 2025

**Authors:** James G. Hodge

**Affiliations:** Sandra Day O’Connor College of Law, Arizona State University, Phoenix, AZ, USA

**Keywords:** public health data, deletion, Executive Order, Constitution, agency

## Abstract

Ongoing efforts among federal agencies to expunge public health data from websites and other media in line with Trump administration directives on “gender ideology” and other themes has led to widespread confusion, angst, and concern among health officials, medical practitioners, and patients. It has also generated legal claims seeking to reverse and stop public health data purges. Framed within statutory or constitutional limits, legal strategies countering these data policies help assure access to core public health information essential to specific services, care, and outcomes.

From the inception of President Trump’s second administration arose a tactic utilized extensively in his first administration — specifically the manipulation and rescission of government information largely for political ends. Following the issuance of a series of Executive Orders (EOs) targeting the transgender community and diversity, equity, and inclusion (DEI) programs, federal agencies were ordered by the Office of Personnel Management (OPM) in late January 2025 to purge their websites and other resources of “gender ideology” and other references. Resulting website redactions through the Department of Health and Human Services (HHS), Centers for Disease Control and Prevention (CDC), Food and Drug Administration (FDA), and other agencies diminished health care services and negatively impacted communal health.

Public health officials, health care workers, researchers, patients, and others reeling from current (and future) pernicious data destructions are taking legal action. An initial lawsuit specifically challenging CDC and FDA has already temporarily restrained public health data purges. Ultimate successes of judicial claims countering politically motivated information redactions, however, are undermined by (1) limitations inherent in a patchwork of federal laws and constitutional uncertainties and (2) the Trump administration’s expressed disdain for adherence to court judgements.^1^

## Scope of Governmental Public Health Data Purges

No one should be surprised by the efforts of the second Trump Administration to purge public health data from federal websites and other resources. Similar efforts were undertaken by President Trump’s first administration, most notably during the COVID-19 pandemic. In support of federal calls to reopen society following weeks of social distancing efforts in the spring of 2020, for example, CDC temporarily ceased posting real-time surveillance data on the spread of the disease to considerable public scorn.^2^ In other ways, as well, the Trump administration repeatedly sought to control governmental information sources from 2016–2020.^3^

What is shocking underlying the current Trump administration efforts is the speed and audacity of its data redactions. Within hours of taking office on Martin Luther King, Jr. Day, January 20, 2025, President Trump issued a series of EOs announcing new prerogatives to federal agencies. Topping the list was the immediate cessation of federal transgender initiatives^4^ and DEI programs.^5^ Corresponding OPM directives clarified the types of agency data purges required via Presidential orders.^6^ “Gender ideology,” “transgender,” “L.G.B.T.,” “inclusion,” “pregnant persons,” and other terms on federal agency websites were expressly prohibited.^7^

Seeking to comply, entire online public health information sources were taken down by January 31. This included thousands of CDC web pages on diverse topics such as assisted reproductive technologies, Alzheimer’s disease treatments, social vulnerabilities in emergencies, and vaccine guidance for pregnant persons.^8^ CDC temporarily deleted its long-standing AtlasPlus surveillance data for HIV, tuberculosis, and other conditions.^9^ Some redacted information sources, like AtlasPlus, were later restored online.^10^ Most were not.

The sweep of public health information deleted from federal sites extended further. As reported by the New York Times,^11^ FDA redacted guidance on assuring diversity of subjects in research trials, the Substance Abuse and Mental Health Services Administration (SAMHSA) removed data on use of the National Disaster Distress Hotline, and the Health Resources and Services Administration (HRSA) stripped information on treating women with opioid addiction. Additional public health data on domestic violence, LGBTQ+ hate crimes, and experimental medicine were struck from other agencies’ websites.

While these initial data purges encompass a mere fraction of available public health information via federal agencies, they represent a disturbing trend incensing medical^12^ and public health organizations.^13^ Initial guidance on data manipulation may lead to further restrictions. Some are concerned that access to the National Institutes of Health (NIH) PubMed^14^ resource and its millions of medical and public health manuscripts may be at risk.^15^ Multiple federal public health agencies, including HRSA and the Administration for Strategic Preparedness and Response (ASPR), were directed by the Trump administration to temporarily stop online posting of new information.

These data policies, coupled with White House withholding of funds for public or private sector health initiatives, contribute to a wholesale diminution of information guiding public health programs, initiatives, and responses. In the face of emerging threats like avian flu and continued rise of anti-vaccine sentiments, lack of information equates to misinformation. And misinformation, as espoused by former FDA Commissioner Robert Califf, contributes to excess morbidity and mortality.^16^ Misinformation of public health data, in short, kills.^17^

## Legal Challenges Addressing Public Health Data Rescissions

Government executive policies contributing to misinformation resulting from redactions of truthful, evidence-based public health data invariably invoke legal objections. Professor Nathan Cortez lists multiple, potential statutory violations extending from data abuses during President Trump’s first term,^18^ including infringements of the Freedom of Information Act (FOIA),^19^ Information Quality Act,^20^ and Whistleblower Protection Act.^21^ “Best available data” statutes, notes Professor Albert Lin, require agencies to make decisions based on available science, but do not typically allow judicial claims for violations.^22^ While these statutory protections may curb data redactions to a degree, Professor Cortez observes how they “fail to reach much of the information mischief of the Trump administration.”^23^

Congress proposed additional barriers to data purges in the Preserving Government Data Act (PGDA) of 2017.^24^ The Act would prohibit federal agencies from blanket deletions of open, available information. Yet the Act also allowed agency directors to expunge data they determined to “not provide sufficient value to the public,” with advance notice.^25^ Such a loose standard could easily support recent data purges backed by Presidential orders. The Scientific Integrity Act, introduced in 2023,^26^ would have prohibited federal agencies from altering or failing to publicly share their scientific findings. Neither bill passed and new legislation directly limiting Presidential data policies is unlikely.

Judicial recourse to counter executive agency data withdrawals may be a last resort, but on what specific grounds? A lawsuit filed just days after initial agency data purges provides potential answers. On February 4, 2025, the nonpartisan medical association, Doctors for America (DFA) sued OPM, HHS, CDC, and FDA in federal district court in Washington, DC.^27^ DFA alleged agency violations of the (1) Administrative Procedures Act (APA),^28^ (2) Paperwork Reduction Act (PRA),^29^ (3) statutory authorities limiting OPM,^30^ and (4) general principles against “arbitrary and capricious” actions. It specifically sought reinstatement of manifold web-related information sources and cessation of further deletions.

On February 11, US District Court Judge John D. Bates issued a temporary restraining order (TRO) in favor of DFA. The court prevented HHS, CDC, and FDA from removing further web information and required renewals of what was previously taken down.^31^ As an initial matter the court found that DFA had associational standing sufficient to bring its claims since some its members were injured by agency data redactions. Whether this determination survives on appeal is questionable given limits of associational standing laid out by the US Supreme Court in *FDA v. Alliance for Hippocratic Medicine* ^32^ in June 2024.

The federal district court further addressed the merits of DFA’s substantive claims of APA and PRA violations. It found that agency removal of public health data from their websites constituted “final agency action,” an essential step to invoke APA violations. It then determined that DFA had a limited right to access government information at least for the interval between when an agency provides notice of its redaction, as required by PRA, and the date of its actual purge. CDC and FDA failures to provide any notice prior to deleting massive health data impinged DFA’s access interests in violation of APA.

## Constitutional Challenges to Federal Data Expulsion Policies

DFA’s obtainment of a TRO is promising, but subject to appeal. DFA could have strengthened its case if it could show federal agencies have a legislative duty to collect and share public health data. It argued that such efforts are consistent with the agencies’ internal, non-binding mission statements. That is not a sufficient basis to compel information sharing.

Ultimately, affected parties may have to strike at the core of federal data policies by challenging the constitutionality of the EOs undergirding mass data rescissions, specifically EOs 14168 (“Gender Ideology”)^33^ and 14151 (“DEI Programs”).^34^ Existing litigation surrounding both orders expose their constitutional deficiencies, although not directly related to their impacts on public health data policy. In *Doe v. McHenry*,^35^ a federal district court judge issued a TRO to prevent the transfers under EO 14168 of several transgender persons from women’s to men’s prisons. The judge relied principally on Eighth Amendment rights against cruel and unusual punishment. In *PFLAG, Inc. v. Trump*, another federal district court judge took similar action to temporarily void prohibitions against providing federal funds to hospitals treating transgender persons under the age 19.^36^

Parties in both cases laid the groundwork for equal protection claims. SCOTUS, which may ultimately adjudge the constitutionality of EO 14168, has favorably interpreted statutory protections assimilating equal protection principles for transgender persons. In *Bostock v. Clayton County*,^37^ the Court determined that discrimination based on sexual orientation or gender identity constitutes sex-based discrimination in violation of Title VII of the Civil Rights Act of 1964.

Equal protection challenges to EO 14151 (DEI Programs) may arise as well. They face, however, a contravening SCOTUS decision to end race-based affirmative action admissions standards in public and private universities.^38^ In 2023 Chief Justice Roberts clarified that collegiate admissions programs may “never use race as a stereotype or negative.”^39^ Similar reasoning applied to government rescissions of DEI programs and accompanying data based in part on race classifications may upend specific equal protection challenges.

To the extent both EOs and administration maneuvers inhibit public access to legitimate, truthful public health data, First Amendment protections may be implicated. If the Trump administration directed private websites to redact “gender ideology” or other similar data, free speech claims would indubitably prevail. Whether First Amendment protections limit government rescission of its own public resources is less certain. For starters, as Professor Eugene Volokh notes, “[g]overnment agencies do not have free speech rights against their own governments.”^40^ Such constitutional claims cannot be brought directly via CDC or FDA employees *per se.* Nor do private associations or others have specific rights to access sensitive or secretive government information (e.g., national security, private medical information, closed proceedings).^41^ Of course, the type of data redacted via recent federal agency actions are not of this ilk. Health-related data sought for reposting were previously publicly available on open websites.

The quintessential, long-standing question is whether Americans have some “fundamental personal right” to access truthful, non-secretive, governmental data denied to them through dubious or arbitrary policies?^42^ SCOTUS has repeatedly rejected such broad claims even as it recognized specific rights to access governmental data among select entities (e.g., media) and settings (e.g., criminal trials).^43^ Professor Mary M. Cheh and others have previously conceptualized a basic, public right to access governmental information via either First Amendment freedoms or structural protections embedded in substantive due process.^44^ It is a forlorn argument most likely lost on a modern, conservative Supreme Court fixated on constitutional limits, as contrasted with fundamental protections.

Still, the time may be ripe for renewed visions on public “right to know” claims to meaningful public health data held via federal agencies. What these agencies withhold under extant orders is problematic. As DFA and other medical and public health associations expressed, governmental rescissions of core public health data impair individual and communal health. The lack of data contributes to public health misinformation tied to political whims.

Even more concerning are the data that federal agencies may be required to withdraw or withhold ahead. The public health repercussions of data manipulations in an environment of evolving threats and questionable national leadership were glimpsed during COVID-19. As noted above, just weeks into the pandemic, federal and state authorities manipulated the types of health data gathered, released, or reported. The consequences were not only unethical, undemocratic, and arguably unconstitutional, but also deadly.
